# Baseline characteristics from CELIA: A Phase 2 study to evaluate BIIB080 in participants with Early Alzheimer’s Disease

**DOI:** 10.1002/alz70859_103364

**Published:** 2025-12-25

**Authors:** Melanie Shulman, Shuang Wu, Jing Xie, Ellen Huang, Amanda Edwards, Jessica Collins, R. Matthew Hutchison, Anne Unwin, Gioacchino Curiale, Guatam Baheti, Martin Jones, Diana Gallagher, Szofia Bullain

**Affiliations:** ^1^ Biogen, Cambridge, MA USA

## Abstract

**Background:**

While the recent availability of amyloid‐targeting disease‐modifying therapies marks a major therapeutic advancement, significant unmet need remains for treatment of Alzheimer’s disease (AD). Aβ deposition is believed to occur early in the pathogenesis of AD, yet clinicopathological studies have established that the density and distribution of tau neurofibrillary tangles are more closely associated with the severity of clinical impairment. Biogen is developing BIIB080, an antisense oligonucleotide (ASO) that inhibits translation of tau mRNAs into protein for the treatment of AD, based on the hypothesis that lowering the production of all tau species will reduce post‐translationally modified forms of tau, including aggregates, leading to slowing or halting of disease progression. Exploratory analyses from the BIIB080 Phase 1b study (NCT03186989) demonstrated a marked effect of BIIB080 on CSF tau and tau PET biomarkers as well as consistently favorable trends in the high‐dose groups on multiple clinical endpoints.

**Method:**

CELIA (NCT05399888) is a randomized, double‐blind, placebo‐controlled, parallel‐group Phase 2 study to further evaluate the efficacy, safety, and tolerability of BIIB080 in participants with early AD. The study enrolled 416 individuals aged 50‐80 years, with MCI or mild AD dementia, Clinical Dementia Rating (CDR) global score of 0.5‐1, Mini Mental State Examination (MMSE) score of 21‐30, objective evidence of cognitive impairment at screening, and confirmed amyloid positivity. Participants were randomized to receive 1 of 3 dose regimens of intrathecal (IT) BIIB080 or placebo every 12 or 24 weeks during the 76‐week placebo‐controlled period. The primary endpoint will assess the dose‐response in change from baseline to week‐76 on the CDR‐SB. The study includes two PET substudies looking at BIIB080 effects: using 18F‐MK‐6240 PET on tau aggregates and using 18F‐florbetapir PET BIIB080 on cerebral amyloid aggregates.

**Result:**

The Phase 2 CELIA study recently completed enrollment. Preliminary review of baseline characteristics demonstrates similarity to those in other recent early AD trials. A full characterization will be presented.

**Conclusion:**

The ongoing Phase 2 CELIA trial will assess the efficacy, safety, and tolerability of BIIB080 compared to placebo in participants with early AD.

